# Machine learning for clinical operations improvement via case triaging

**DOI:** 10.1002/ski2.83

**Published:** 2021-12-15

**Authors:** S. J. Huang, Y. Liu, K. Kanada, G. S. Corrado, D. R. Webster, L. Peng, P. Bui, Y. Liu

**Affiliations:** ^1^ Google Health via Advanced Clinical Deerfield Illinois USA; ^2^ Google Health Palo Alto California USA

Dear Editor,

In recent years, an increasing number of machine learning (ML) models have been developed for interpreting images of skin conditions^1^, ^2^ and for risk stratification.^3^, ^4^ Beyond accurate image interpretation, one potential application of these interpretations may be triaging systems to help direct care to the right care provider at the right time.^5^, ^6^ This is a critical need because dermatologist appointment wait times exceed a month in many regions,^7^, ^8^ a trend that can potentially be alleviated by rapidly stratifying patients to clinicians with the appropriate level of training (e.g., board‐certified dermatologist, advanced practice provider under dermatologist supervision, non‐dermatologist) and the appropriate urgency.

To help understand ML's potential for this triaging, we analysed a previously‐described deep learning system (DLS) that provides a differential diagnosis of teledermatology cases^1^ and that improved the diagnostic accuracy of primary care physicians and nurse practitioners in a randomized study.^2^ Specifically, we (K.K.) mapped each of the DLS's 419 skin conditions to 1 of 5 clinical triage categories ranging from ‘immediate intervention is needed’ to ‘no need to see a doctor’ (Figure 1). These determinations were based on clinical judgement, assuming the worst case clinical outcome possible from delaying care leading to prolonged lack of diagnosis and treatment. For the validation dataset of the original study^1^ (*3756* consecutive visits; see Validation set A in Table 1 of the original manuscript), we then defined the urgency of each case based on the urgency categorisation of the primary differential diagnosis of a panel of dermatologists. After excluding cases for which there were two most‐likely diagnoses from the panel and where the two diagnoses mapped to different urgency levels (and thus each case's urgency was ambiguous), *3494* visits remained.

**FIGURE 1:**
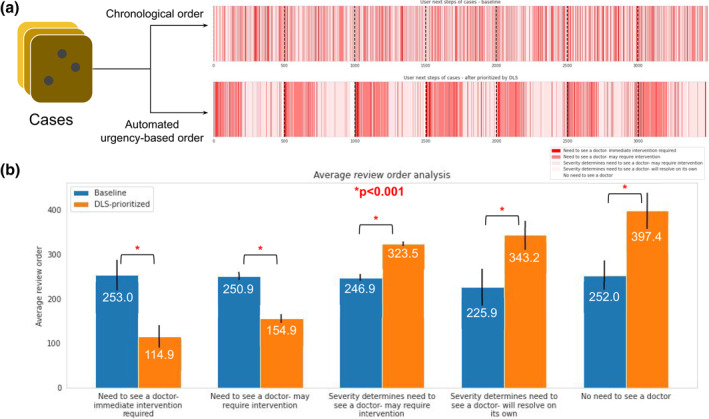
Effect of reordering batches of teledermatology cases using the deep learning system (DLS)'s top predicted differential diagnosis. (a) Top reflects the original chronological ordering of cases from left to right, with red lines indicating urgent cases (based on a dermatologist panel's top differential diagnosis), lighter‐red indicating less urgent cases, and white indicating cases that do not need dermatologist attention. Bottom reflects the reordered set of 3494 cases in batches of 500, based on the DLS's predicted differential. (b) Comparison of average ordering of cases between original chronological order (blue) versus the DLS‐triaged order (orange). Blue bars are close to 250 (out of 500) across all case categories, indicating random ordering, whereas orange bars are lower for urgent cases and higher for less urgent cases. Error bars indicate the standard deviation of the average rank across all batches and *p*‐values from a permutation test

Next, we grouped these teledermatology cases into ‘review batches’ of 500 (Figure 1a) and computed the average review order (i.e., if two cases were reviewed first and fifth, the average order would be three) for cases in each ‘urgency category’ (Figure 1b). As expected, the consecutive order in which the cases arrived resulted in all categories being reviewed in a random order (average rank ≈250 of 500); cases that likely did not need physician attention were seen just as quickly as urgent cases that needed immediate intervention.

To understand if the DLS could help, we next reordered the cases within each ‘review batch’ of 500 based on the urgency category of the DLS‐predicted skin condition (which is an automated process requiring no human intervention). On average, this caused the review order of urgent cases to be prioritised substantially sooner than that of less urgent cases, with the average rank of ‘immediate intervention cases’ being about 100 (vs. 253 without reordering, *p* < 0.001), and that of ‘no need to see a doctor’ cases being close to 400 (vs. 252 without reordering, *p* < 0.001).

These results demonstrate the potential for how a teledermatology practice could productively leverage a ML model to prioritise cases for review and the efficacy of such an automated triage system. The specific actions from these insights will likely depend on the operational expertise and preferences of the practice. For example, the average turnaround‐time could be improved for urgent cases requiring immediate intervention by flagging them for immediate review and follow‐up, or cases could be triaged to the appropriate type of care setting (e.g., emergency room vs. urgent care vs. clinic). Beyond teledermatology case prioritisation, these insights may be valuable to dermatology practices that implement an electronic consult system via photograph submission directly from patients or from other providers. At some health systems, tele‐dermatologists and/or dermatology trainees triage the urgency of cases.^9^, ^10^ Our approach would be an automated version of these tele‐dermatologist triage systems, with the potential to accelerate triaging and reduce the burden on the limited dermatology workforce to focus on patient management.

Our proof‐of‐concept triaging application has limitations and room for future improvement, both in terms of training data diversity^1^ and the modelling approach. For example, instead of inferring the urgency level from its skin condition output, developing a DLS to specifically predict case urgency could lead to improved triage accuracy. We could also do further studies to triage cases to a specific type of provider (e.g., dermatologist, primary care physician, advanced practice provider, etc) or care location. Practices also often have their own varying approaches for triage (including the referring clinician flagging certain cases for expedited review); understanding how each practice could apply their own rules to our DLS to suit their individual needs would be useful. Finally, further research will be needed to test such a triage system in an actual clinic setting for feedback and to assess for gaps. For example, cases that present with multiple conditions may need to be assigned the higher priority amongst conditions, as a safety measure.

To conclude, we have presented a preliminary study on leveraging ML to address a key clinical operations problem: triaging to identify and potentially see urgent cases more quickly, and hope to inspire additional projects using ML to address important clinical operational issues.

## FUNDING INFORMATION

Google LLC.

## CONFLICT OF INTEREST

Most authors are employees of Google and own Alphabet stock.

## AUTHOR CONTRIBUTIONS

**S. J. Huang:** Conceptualization; Data curation; Writing – original draft; Writing – review & editing. **Y. Liu:** Conceptualization; Formal analysis; Investigation; Methodology; Supervision; Visualization; Writing – original draft; Writing – review & editing. **K. Kanada:** Data curation; Formal analysis; Investigation; Methodology; Writing – review & editing. **G. S. Corrado:** Funding acquisition; Project administration; Supervision; Writing – review & editing. **D. R. Webster:** Conceptualization; Funding acquisition; Project administration; Supervision; Writing – review & editing. **L. Peng:** Conceptualization; Formal analysis; Project administration; Supervision; Visualization; Writing – review & editing. **P. Bui:** Conceptualization; Data curation; Funding acquisition; Project administration; Writing – review & editing. **Y. Liu:** Conceptualization; Data curation; Formal analysis; Funding acquisition; Investigation; Methodology; Project administration; Resources; Software; Supervision; Validation; Visualization; Writing – original draft; Writing – review & editing.

## Data Availability

The dataset is not publicly available owing to privacy considerations.
